# Catastrophic Failure and Critical Scaling Laws of Fiber Bundle Material

**DOI:** 10.3390/ma10050515

**Published:** 2017-05-09

**Authors:** Shengwang Hao, Hang Yang, Xiangzhou Liang

**Affiliations:** 1 School of Civil Engineering and Mechanics, Yanshan University, Qinhuangdao 066004, China; yh928@stumail.edu.cn (H.Y.); liangxiangzhou@foxmail.com (X.L.); 2 The State Key Laboratory of Nonlinear Mechanics, Institute of Mechanics, Chinese Academy of Science, Beijing 100190, China

**Keywords:** catastrophic failure, scaling law, fiber, fiber bundle model, heterogeneous material

## Abstract

This paper presents a spring-fiber bundle model used to describe the failure process induced by energy release in heterogeneous materials. The conditions that induce catastrophic failure are determined by geometric conditions and energy equilibrium. It is revealed that the relative rates of deformation of, and damage to the fiber bundle with respect to the boundary controlling displacement *ε*_0_ exhibit universal power law behavior near the catastrophic point, with a critical exponent of −1/2. The proportion of the rate of response with respect to acceleration exhibits a linear relationship with increasing displacement in the vicinity of the catastrophic point. This allows for the prediction of catastrophic failure immediately prior to failure by extrapolating the trajectory of this relationship as it asymptotes to zero. Monte Carlo simulations are completed and these two critical scaling laws are confirmed.

## 1. Introduction

Material damage and fracture has attracted a large amount of theoretical and experimental interest owing to their relationship to many failure phenomena occurring in naval, aeronautics, and space industries [1], as well as the damage occurring due to earthquakes [2,3,4]. The underlying microscopic mechanism of failure is so complex that it is far from being well understood.

Localization is a common phenomenon appearing in the evolution of strain (or damage) that ultimately induces material failure and it is a significant factor in the complexity of fracture. After localization, a sample will bifurcate into a two-part continuum consisting of a less-deformed zone plus a highly-deformed (damage) band called a localized zone [5,6,7,8]. The localized zone is mechanically and physically distinct from the surrounding zones. This implies that when the eventual macroscopic failure occurs, the scale governing the macroscopic failure is much smaller than that of the sample size [8,9]. Catastrophic failure occurs when the energy released from either the testing system or from outside the localized zone (or both) can compensate for the required fracture energy of the localized zone [8,9,10].

Analyzing the precursors to failure has been a long-standing problem and has been widely accepted as a significant way to predict material failure [5,11,12,13,14,15,16]. Voight [12,13] proposed a materials failure law to describe the accelerating precursory immediately prior to failure. The Materials Failure Forecasting Method (FFM) [5,14,15,16,17,18], which is based on the accelerating precursors, has been proposed for the prediction of natural disasters such as volcanic eruptions, earthquakes and landslides.

In materials science and engineering, the well-known fiber bundle models (FBM), which is a class of simple models, has been widely used [18,19,20,21] to explain the failure mechanism of materials ever since Peirce [22] first developed this model to study the strength of cotton yarn. This model has proven to be very effective in practical applications and various aspects of failure in composite materials such as fiber reinforced composites and other disordered materials [18,23,24,25,26]. Because a study of the strength properties of certain materials usually needs to involve considerations fundamentally similar to those arising in fiber bundle theory [19,22], this model has been increasingly used to explain failure processes in many other heterogeneous materials [21,27].

In this paper, a model composed of an elastic spring and a fiber bundle oriented in series is developed to describe the catastrophic failure of a material induced by the energy release from the system. The conditions for catastrophic failure are derived based on energy equilibrium. An asymptotic analysis is used to derive the critical scaling laws near the catastrophic point. Monte Carlo simulations are performed to verify two critical scaling laws describing the failure.

## 2. Model Description

**Figure 1:**
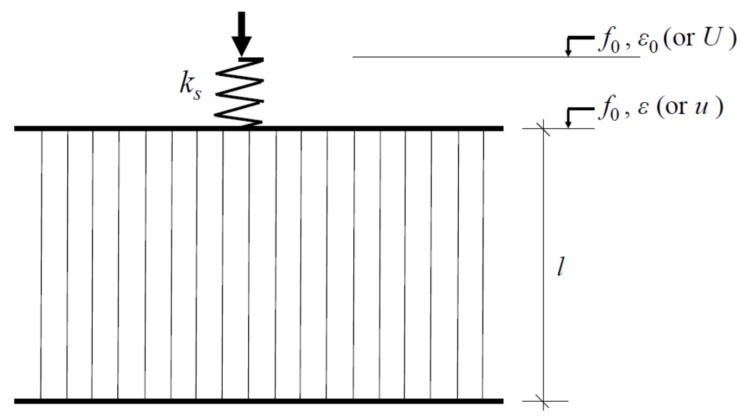
Sketch of the spring-fiber bundle model. *f*_0_: Normalized resulting force; *U*: Boundary displacement, *ε*_0_ = *U*/*l*; *u:* Deformation of the fiber bundle, *ε = u*/*l*.

In order to demonstrate the catastrophic failure induced by the energy release, a system consisting of a linear elastic spring and a damageable part oriented in series, as shown in Figure 1, is put under focus. In this paper, the boundary displacement and the deformation of the damageable part are denoted by *U* and *u*, respectively. The spring of stiffness *k*_s_ can be analogous to an elastic environment (such as the load apparatus or the zones outside the localized zone). The damageable part consists of *N* parallel fibers with a linearly elastic constitutive behavior. A global load-sharing criterion [20,21,23,24] is chosen for the load redistribution following the break of one or more of the fibers. From this form, some closed analytic results can be obtained. All the fibers are assumed to have the same stiffness until they break. A fiber breaks when it reaches its strength, and thus no longer carries any load. The surviving fibers equally share the force released by the broken fibers.

Before it breaks, a fiber follows a linearly elastic constitutive behavior, given as:

(1) $$k_{d}\;u=p$$

where *p* is the force on a fiber element and *k_d_* denotes the stiffness of the individual fiber element. The strain is given by *ε* = *u*/*l* where *l* is the length of a fiber. Equation (1) is then rewritten as:

(2) ε=f,

where *f*_0_ = *p*/(*lk_d_*) is the dimensionless true force on an individual intact fiber element. The resulting force on the system is *F* = *Np,* which is normalized as *f*_0_ = *F*/(*Nlk_d_*).

The resulting force acting on the spring can also be expressed as *F* = *k*_s_*u*_s_, where *u*_s_ is the deformation of the elastic spring. The geometric condition of Figure 1 implies that the deformation of the elastic spring can be written as *u*_s_ = *U* − *u*, resulting in:

*f*_0_ = *k*(*ε*_0_ − *ε*), (3)

where *ε*_0_ = *U*/*l* is the normalized boundary displacement. *k* = *k*_s_/(*Nk_d_*) represents the initial stiffness ratio of the elastic spring to that of the damageable part. At a certain strain *ε* for the fibers in the damageable part, the true load on every surviving fiber is *f* = *f*_0_/[1 − *D*(*ε*)], where *D*(*ε*) = *N*_d_/*N* represents the damage fraction of damageable part and *N*_d_ is the number of broken fibers. It is clear that the damage fraction, *D*, ranges from zero to unity. The force-deformation relation of the damageable part can then be written as:

*f*_0_ = [1 − *D*(*ε*)]*ε* (4)

For the system to be in the equilibrium, Equations (3) and (4) must be equal, such that:

*k*(*ε*_0_ – *ε*) = [1 − *D*(*ε*)]*ε* (5)

The damage fraction is widely described by a Weibull distribution of the form $D\left(\varepsilon \right)=1-e^{-\varepsilon^{m}}$ [11,17,18,19], where *m* is the Weibull index.

## 3. Critical Condition that Induces Catastrophic Failures

The equilibrium of the spring-fiber bundle system becomes unstable when the work needed for further deformation of the damageable part (Δ*W*_c_) can be fully provided by the energy release of the spring (Δ*W*_s_) without any external work, thus allowing for breaking (or deformation) to continue spontaneously and uncontrollably. In other words, the equilibrium is stable if:

Δ*W*_s_ < Δ*W*_c_ (6)

The work done by the elastic spring during virtual deformation Δ*ε*_s_ is:

Δ*W*_s_ = (*f*_0_ + 1/2Δ*f*_0_)·Δ*ε*_s_, (7)

where *ε*_s_ represents the normalized deformation of the spring.

The energy required to impose a similar deformation (Δ*W*_d_) by an increment of Δ*ε* on the damageable part is:

Δ*W*_d_ = (*f*_0_ + 1/2Δ*f*_0_)·Δ*ε* (8)

Substituting Equations (7) and (8) into Equation (6) gives:

−Δ*ε*_s_ = Δ*f*_0_/*k* < Δ*ε* = Δ*f*_0_/(d*f*_0_/d*ε*) (9)

The negative value of Δ*ε*_s_ implies that the elastic spring undergoes a deformation recovery process. Expression (9) leads to *k* > −d*f*_0_/d*ε*. Therefore, the critical condition that induces catastrophic failure is:

*k* = −d*f*_0_/d*ε* (10)

From Equations (4), (5) and (10), the critical condition can also be expressed as:

(d*ε*_0_/d*ε*)_f_ = 0 (11)

Thus, no macroscopic failure occurs when *k* > *k*_c_ = −(d*f*_0_/d*ε*)_min_, where (d*f*_0_/d*ε*)_min_ represents the minimum value of the tangent slope of the *f*_0_-*ε* curve of the fiber bundle. The deformation can be analytically derived as $\varepsilon ={\left(1+1/m\right)}^{1/m}$ through a Weibull distribution by setting $d^{2}f_{0}/d\varepsilon^{2}=0$ at the point of (d*f*_0_/d*ε*)_min_, resulting in $k_{c}=me^{-\left(1+1/m\right)}$.

**Figure 2:**
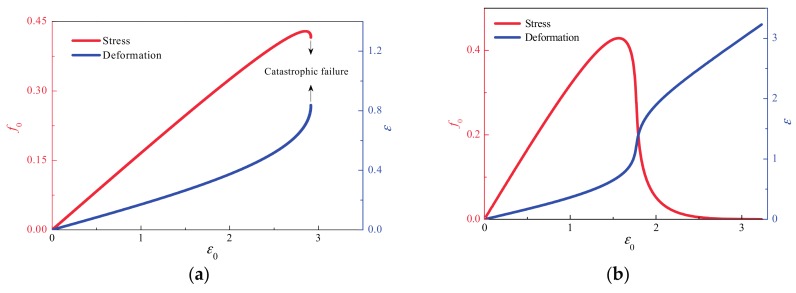
Analytical results of force and deformation versus displacement for *k* > *k*_c_ and *k* < *k*_c_ when *m* = 2, *N* = 10^4^ and k_c_ = 0.446. (**a**) *k* = 0.2 (i.e., *k* < *k*_c_). The catastrophic failure occurs following the peak force; (**b**) *k* = 0.5 (i.e., *k* > *k*_c_). The force continuously decreases to zero and thus no catastrophic failure occurs during the loading process.

As examples, Figure 2 illustrate the change in force and deformation with increasing boundary displacement (ε_0_) for *k* < *k*_c_ and *k* > *k*_c_. In these two cases, the Weibull index m equals 2, resulting in a *k*_c_ value of about 0.446. It can be seen that when *k* = 0.2 (that is, *k* < *k*_c_), the catastrophic failure occurs following the peak force (Figure 2a). In contrast, when *k* = 0.5 (that is, *k* > *k*_c_), the stress decreases continuously to zero and the failure is not catastrophic but gradual, as shown in Figure 2b.

## 4. Critical Scaling Law near the Point of Catastrophic Failure

An asymptotic analysis of the area in the vicinity of the catastrophic point to demonstrate the critical scaling law is not presented. The geometric Equation (5) implies that the boundary displacement *ε*_0_ can be expressed as a function of the deformation *ε* such that *ε*_0_ = [1 − *D*(*ε*)]*ε*/*k* + *ε*. The expansion of *ε*_0_ as a function of *ε* in the vicinity of the catastrophic point *ε*_0f_ is then performed. That is:

(12) $$\varepsilon_{0}\approx \varepsilon_{0f}+{\left(\frac{d\;\varepsilon_{0}}{d\varepsilon }\right)}_{f}\left(\varepsilon_{f}-\varepsilon \right)+\frac{1}{2}{\left(\frac{d^{2}\varepsilon_{0}}{d\varepsilon^{2}}\right)}_{f}{\left(\varepsilon -\varepsilon_{f}\right)}^{2}$$

Substituting Equation (11) into Equation (12), we get:

(13) $$\varepsilon \approx \varepsilon_{f}-{\left[-\frac{1}{2}{\left(d^{2}\varepsilon_{0}/d\varepsilon^{2}\right)}_{f}\right]}^{-\frac{1}{2}}{\left(\varepsilon_{0f}-\varepsilon_{0}\right)}^{\frac{1}{2}}$$

By performing the first differentiation on Expression (13) with respect to *ε*_0_, the following relation is obtained:

(14) $$d\varepsilon /d\varepsilon_{0}∼{\left(\varepsilon_{0f}-\varepsilon_{0}\right)}^{\frac{1}{2}}.$$

Therefore, the deformation rate of the damageable part increases under a power law behavior with an exponent of −1/2 near the catastrophic point.

Furthermore, by performing the first and second differentiation on Expression (13) with respect to *ε*_0_ and rearranging, the following relation is obtained:

(15) $$\frac{d\varepsilon }{d\;\varepsilon_{0}}{\left(\frac{d^{2}\varepsilon }{d\;{\varepsilon_{0}}^{2}}\right)}^{-1}=2(\varepsilon_{0f}-\varepsilon_{0})$$

An analogous procedure may be applied to calibrate in terms of damage *D* by noting that the deformation can be expressed as a function of *D*. For example, $\varepsilon ={\left[-\log(1-D)\right]}^{1/m}$ for a Weibull distribution. Then, similar expressions can be deduced as:

d*D*/d*ε*_0_ ~ (*ε*_0f_ − *ε*_0_)^−1/2^ (16)

and:

(17) $$\frac{d\;D}{d\;\varepsilon_{0}}{\left(\frac{d^{2}D}{d\;{\varepsilon_{0}}^{2}}\right)}^{-1}=2(\varepsilon_{0f}-\varepsilon_{0})$$

**Figure 3:**
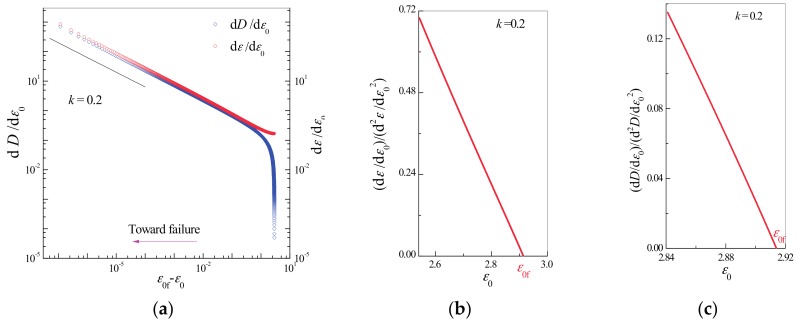
Critical scaling laws in the vicinity of the catastrophic point for the case shown in Figure 2b. (**a**) Critical power law behaviors of d*ε*/d*ε*_0_ ~ (*ε*_0f_ − *ε*_0_)^−1/2^ and d*D*/d*ε*_0_ ~ (*ε*_0f_ − *ε*_0_)^−1/2^. A straight line of slope −1/2 is drawn to guide the eye. Relationship of (**b**) d*ε*/d*ε*_0_ (d^2^*ε*/d*ε*_0_^2^)^−1^ and (**c**) d*D*/d*ε*_0_(d^2^*D*/d*ε*_0_^2^)^−1^ to displacement *ε*_0_ near the catastrophic failure point. The approximately linear relationship in the vicinity of the catastrophic point *ε*_0f_ (with a slope of 2) verifies the relationship d*ε*/d*ε*_0_ (d^2^*ε*/d*ε*_0_^2^)^−1^ = 2 (*ε*_0f_ − *ε*_0_) and d*D*/d*ε*_0_ (d^2^*D*/d*ε*_0_^2^)^−1^ = 2 (*ε*_0f_ − *ε*_0_).

To examine Relations (14), (15), (16), and (17), the first and second derivatives of damage *D* and strain *ε* are calculated with respect to the controlling variable $\varepsilon_{0}$ resulting in $d\varepsilon /d\varepsilon_{0}$ (or $dD/d\varepsilon_{0}$) and $d^{2}\varepsilon /d{\varepsilon_{0}}^{2}$ (or $d^{2}D/d{\varepsilon_{0}}^{2}$) for *m* = 2. Based on the observed linear dependence (the left part of the curves in Figure 3a), the increase in deformation near the catastrophic failure point conforms to the power law relations given in (14) and (16). The linear relationship with a slope of 2 between $d^{2}\varepsilon /d{\varepsilon_{0}}^{2}$ (or $d^{2}D/d{\varepsilon_{0}}^{2}$) and $\varepsilon_{0}$ in the vicinity of the catastrophic failure point shown in Figure 3b,c confirms the veracity of Relations (15) and (17). Based on Relations (15) and (17), the catastrophic point can be predicted as the intersection point of the abscissa axis with the linear extrapolation of the curve of d*ε*/d*ε*_0_(d^2^*ε*/d*ε*_0_^2^)^−1^ (or d*D*/d*ε*_0_(d^2^*D*/d*ε*_0_^2^)^−1^) against *ε*_0_ to zero.

## 5. Numerical Analysis

To further examine the critical behaviors near the catastrophic failure point, Monte Carlo simulations of the failure process were performed. Simulations of the failure process proceeded as follows: as the displacement on the system with a fiber bundle of *N* fibers monotonically increased, (1) fiber breaking thresholds were randomly chosen according to the Weibull probability distribution with the thresholds then arranged in increasing order; (2) The load process was performed quasi-statically with a displacement of *ε*_0_ applied at each step as the minimum required to break the next fiber. After the breakage of a single fiber, the nominal force *f*_0_ on the system and consequently the deformation *ε* of the fiber bundle were recalculated. This process was repeated until the load on all surviving fibers was less than that of their individual thresholds; (3) The system is then loaded again and the process is repeated until the material fails in its entirety. During step 2, the break of an element may induce secondary failures which may in turn trigger more failures, and so on. If this occurs, this process will lead to a catastrophic failure.

**Figure 4:**
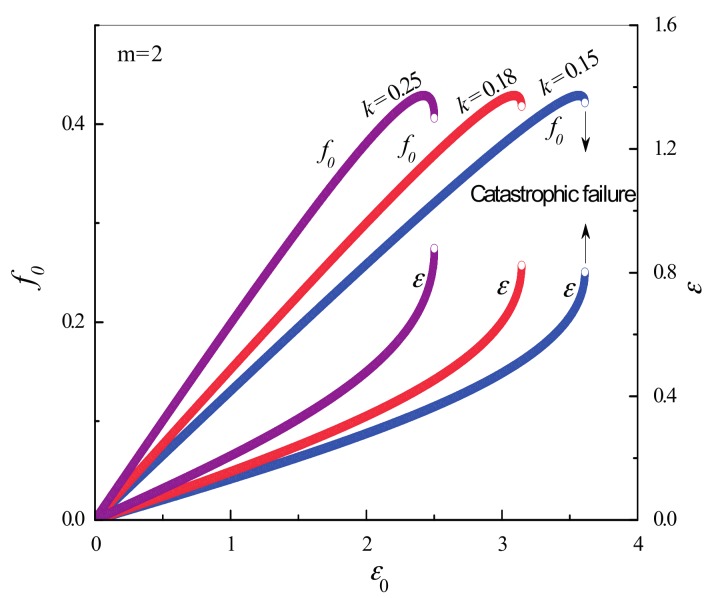
Simulation results of *f*_0_ − ε_0_ curves with *m* = 2.

**Figure 5:**
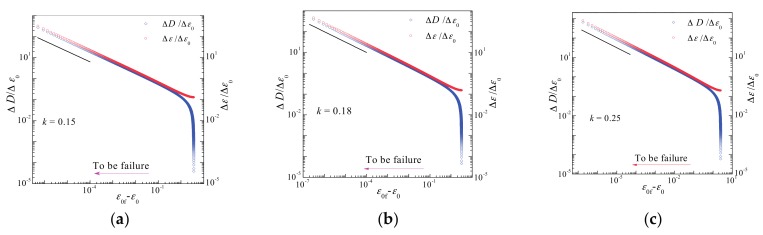
Critical power law behaviors in the vicinity of the catastrophic failure point for the case shown in Figure 2b. (**a**) *k* = 0.15; (**b**) *k* = 0.18; (**c**) *k* = 0.25. The straight line of slope −1/2 is drawn to guide the eye.

**Figure 6:**
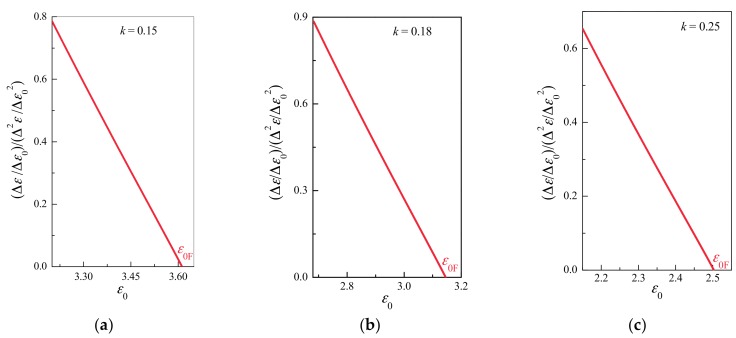
Critical relationship between d*ε*/d*ε*_0_(d^2^*ε*/d*ε*_0_^2^)^–1^ and displacement *ε*_0_ near the catastrophic failure point for the cases shown in Figure 4. (**a**) *k* = 0.15; (**b**) *k* = 0.18; (**c**) *k* = 0.25. An almost linear relationship between d*ε*/d*ε*_0_(d^2^*ε*/d*ε*_0_^2^)^–1^ and *ε*_0_ was exhibited by all samples in the vicinity of the catastrophic failure point.

**Figure 7:**
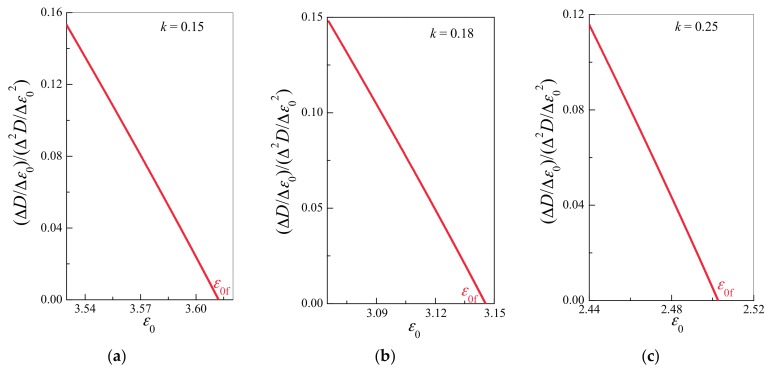
Critical relationship between d*D*/d*ε*_0_(d^2^*D*/d*ε*_0_^2^)^−1^ and displacement *ε*_0_ near the catastrophic failure point for the cases shown in Figure 4. (**a**) *k* = 0.15; (**b**) *k* = 0.18; (**c**) *k* = 0.25. An almost linear relationship between d*D*/d*ε*_0_(d^2^*D*/d*ε*_0_^2^)^−1^ and *ε*_0_ was exhibited by all samples in the vicinity of the catastrophic failure point.

Figure 4, Figure 5, Figure 6 and Figure 7 illustrate the simulation results for three samples for different *k* values. As shown in Figure 5, damages and deformation for all samples exhibit common critical power law behaviors of d*ε*/d*ε*_0_ ~ (*ε*_0f_ − *ε*_0_)^−1/2^ and d*D*/d*ε*_0_ ~ (*ε*_0f_ − *ε*_0_)^−1/2^ with a critical exponent of −1/2. In all simulations, ∆*ε*/∆*ε*_0_(∆^2^*ε*/∆*ε*_0_^2^)^−1^ and ∆*D*/∆*ε*_0_(∆^2^*D*/∆*ε*_0_^2^)^−1^ exhibit a common linear relationship under a displacement of *ε*_0_ near the catastrophic failure point (see Figure 6 and Figure 7), even though their failure displacements are different (see Figure 4). In the discrete cases of simulations, the discrete derivative operator is denoted “∆”, as opposed to the continuous notion of derivative “d”.

## 6. Discussion

### 6.1. Models of Catastrophic Failure Induced by Energy Release

In the laboratory tests of the heterogeneous materials, a sample is usually loaded by monotonically increasing the displacement of the testing machine crosshead [7,28,29]. The loading apparatus deforms associated with the deformed sample and thus stores the elastic strain energy. The loading apparatus will release the stored energy through the recovery of the deformation during the strain-softening phase after the peak force. When the energy release of the loading apparatus can compensate for the fracture energy of the sample, the failure becomes self-sustaining without the need of additional external work, and thus becomes catastrophic [7,28,29].

Many catastrophic events such as the instability of pillars in mining engineering [30], earthquakes, rock outbursts, and avalanches are driven by mechanisms similar to those discussed in this paper, and are explained by models [28,30,31,32,33] similar to the elastic-damageable part model presented in this paper. The elastic spring has always been used to represent tributary zones such as a loading apparatus, the zones outside the localized zone, and the rock mass surrounding faults and pillars. A famous example is the spring-slider model that is used to demonstrate the stick-slip mechanism of faults [31,32,33].

### 6.2. Critical Scaling Laws and their Application in Failure Prediction

The accelerating precursory signals near the material failure point represent a practical basis for the application of failure forecasting models. Many catastrophic events, such as the collapse of engineering structures, natural catastrophes and abrupt weather changes, all share similar critical scaling laws [1,2,15,16]. In many current models for precursory acceleration, the rate of an observable quantity Ω is usually described by an empirical relationship [1,2,3,4,11,12,13,14,15,16,20,21,22,23,24,25,26]:

(18) $$\dot{\Omega }=C{\left(t_{f}-t\right)}^{-\beta }$$

where *t*_f_ is the failure time, *C* is a scaling parameter, and *β* is the critical exponent.

Equation (18) is obtained mainly based on empirical analyses of creep deformation under constant load. However, in practical engineering, materials are usually subject to different loading conditions besides creep deformation. In considering that creep is not the dominant factor of material failure, Kilburn [34] proposed a model to extend analyses to deformations under increasing stress by accommodating changes with stress. He suggested an alternative expression to describe how precursory time series can be determined from a relation between fracturing and stress. Hao et al. [28] introduced a response function as the change in the deformation of the sample with respect to the crosshead displacement of a testing machine and found that the response function showed a critical power-law singularity at the failure point. In the tests, the crosshead displacement is a combination of the deformations of both the loading apparatus and the deformed sample.

The empirical Equation (18) is usually restricted to describe stress-rate-dependent material failure resulting in precursory rates being measured with respect to time. For a “stress-rate independent” material, which is defined as the case where stress in the material is independent of the strain rate, the conditions for material failure are not immediately evident from using time variations alone. The deformation and damage primarily depend on the controlling variable such as, for example, the boundary displacement shown in the present model in this paper. For these kinds of materials, the relative change of measurable responses such as damage and strain, with respect to the controlling variable, are most useful in its application. Hao et al. [11] proposed a similar precursory relation by defining the response function as the relative change of measurable responses (such as damage and strain) with respect to the controlling stress when the material is subject to a monotonically increasing stress.

Equation (1) is of widespread interest as a forecasting tool and has been extensively applied to material failure phenomena. Equations (14) and (16) are equivalent to the relation given in (18) if the boundary displacement *ε*_0_ is increased at a constant rate with respect to time such that d*ε*/d*ε*_0_ (d*D*/d*ε*_0_) ∝ d*ε*/d*t* (d*D*/d*t*). This result may suggest that the time-derivatives given in Relation (18) might be a subset of a more general expression connecting the controlling variable derivatives.

In application, Equations (14) and (16) would be rewritten into a linear form, given as:

(19) $${\left(d\;R/d\;\varepsilon_{0}\right)}^{-1/\beta }=k^{-1/\beta }(\varepsilon_{0f}-\varepsilon_{0}),$$

where *R* represents a corresponding response variable (such as the strain and damage discussed in this paper). The failure point can then be determined by linearly extrapolating the curve of (d*R*/d*ε*_0_)^–1/^^β^ against *ε*_0_ to zero. In comparison, the prediction made by using Relations (15) or (17) does not have the benefit of knowing in advance the value of the exponent *β*. The failure time can be estimated by linearly extrapolating the curve of the proportion of the signal rate on the acceleration against the controlling variable, the boundary displacement *ε*_0_, to zero. In the application of these two methods, it is both feasible and preferable that the two methods are used together in order for each one to verify the trend given by the other.

It should be mentioned that for the present method (Equations (15) and (17)), the signal has to be differentiated with respect to the boundary displacement and then inverted. These calculations will inevitably induce large fluctuations and thus pose an important limitation in terms of real-time operational usage. Two methods for point data and continuous deformation data, respectively, were suggested by Bell et al. [35,36] to diminish such a limitation by the use of a likelihood function and a Global Linearized Model (GLM) of the unprocessed signal rate.

## 7. Conclusions

A model of a spring-fiber bundle oriented in series is proposed to describe material failure. Two failure processes are observed. The first process is the gradual failure characterized by a continuous force that decreases to zero. The second is a catastrophic failure characterized by a violent avalanche of fibers. A critical condition inducing the catastrophic failure is reached when the stiffness of the elastic spring is equal to the negative tangent slope of the force-deformation curve of the fiber bundle. Thus, the catastrophic failure will not occur at any point during the loading process if the stiffness of the spring is larger than the −(d*f*_0_/d*ε*)_min_ of the minimum slope of the force-deformation curve of the damageable part.

Two critical scaling laws work as the precursors of catastrophic failure. The deformation rate of the damageable part increases according to the power law behavior $d\varepsilon /d\varepsilon_{0}∼{\left(\varepsilon_{0f}-\varepsilon_{0}\right)}^{-1/2}$ near the catastrophic failure point. $\frac{d\varepsilon }{d\;\varepsilon_{0}}{\left(\frac{d^{2}\varepsilon }{d\;{\varepsilon_{0}}^{2}}\right)}^{-1}$ (or $\frac{d\;D}{d\;\varepsilon_{0}}{\left(\frac{d^{2}D}{d\;{\varepsilon_{0}}^{2}}\right)}^{-1}$) presents a linear relationship with $\left(\varepsilon_{0f}-\varepsilon_{0}\right)$. This suggests that the catastrophic point $\varepsilon_{0f}$ can, potentially, be predicted by a linear extrapolation of the curve of $\frac{d\varepsilon }{d\;\varepsilon_{0}}{\left(\frac{d^{2}\varepsilon }{d\;{\varepsilon_{0}}^{2}}\right)}^{-1}$ (or $\frac{d\;D}{d\;\varepsilon_{0}}{\left(\frac{d^{2}D}{d\;{\varepsilon_{0}}^{2}}\right)}^{-1}$) against $\varepsilon_{0}$ to zero.
